# QuantImage v2: a comprehensive and integrated physician-centered cloud platform for radiomics and machine learning research

**DOI:** 10.1186/s41747-023-00326-z

**Published:** 2023-03-22

**Authors:** Daniel Abler, Roger Schaer, Valentin Oreiller, Himanshu Verma, Julien Reichenbach, Orfeas Aidonopoulos, Florian Evéquoz, Mario Jreige, John O. Prior, Adrien Depeursinge

**Affiliations:** 1 Institute of Informatics, School of Management, HES-SO Valais-Wallis, Sierre, Switzerland; 2 Department of Oncology, Precision Oncology Center, Lausanne University Hospital and University of Lausanne, Lausanne, Switzerland; 3 Department of Nuclear Medicine and Molecular Imaging, Lausanne University Hospital and University of Lausanne, Lausanne, Switzerland; 4 Knowledge and Intelligence Design Group, Delft University of Technology, Delft, The Netherlands

**Keywords:** Artificial intelligence, Biomarkers, Cloud computing, Decision support techniques, Radiomics

## Abstract

**Background:**

Radiomics, the field of image-based computational medical biomarker research, has experienced rapid growth over the past decade due to its potential to revolutionize the development of personalized decision support models. However, despite its research momentum and important advances toward methodological standardization, the translation of radiomics prediction models into clinical practice only progresses slowly. The lack of physicians leading the development of radiomics models and insufficient integration of radiomics tools in the clinical workflow contributes to this slow uptake.

**Methods:**

We propose a physician-centered vision of radiomics research and derive minimal functional requirements for radiomics research software to support this vision. Free-to-access radiomics tools and frameworks were reviewed to identify best practices and reveal the shortcomings of existing software solutions to optimally support physician-driven radiomics research in a clinical environment.

**Results:**

Support for user-friendly development and evaluation of radiomics prediction models via machine learning was found to be missing in most tools. QuantImage v2 (QI2) was designed and implemented to address these shortcomings. QI2 relies on well-established existing tools and open-source libraries to realize and concretely demonstrate the potential of a one-stop tool for physician-driven radiomics research. It provides web-based access to cohort management, feature extraction, and visualization and supports “no-code” development and evaluation of machine learning models against patient-specific outcome data.

**Conclusions:**

QI2 fills a gap in the radiomics software landscape by enabling “no-code” radiomics research, including model validation, in a clinical environment. Further information about QI2, a public instance of the system, and its source code is available at https://medgift.github.io/quantimage-v2-info/.

**Key points**

- As domain experts, physicians play a key role in the development of radiomics models.
- Existing software solutions do not support physician-driven research optimally.
- QuantImage v2 implements a physician-centered vision for radiomics research.
- QuantImage v2 is a web-based, “no-code” radiomics research platform.

## Background

The widespread availability of digital medical imaging, advances in information technology, and the shift of clinical medicine toward increasingly personalized care have given rise to the exponential growth of a new field of image-based medical biomarker research over the past 10 years [1]: “radiomics.” This approach is based on the premise that medical images contain information about the underlying pathophysiology which, even if not visible to the human eye, can be captured via quantitative image analysis [2]. In contrast to traditional image analysis that focuses on visual interpretation, “classical” radiomics analysis involves the automated high-throughput conversion of routine clinical images into mineable data collections of so-called “agnostic” features that provide a quantitative description of the shape, intensity distribution, and texture of an image region of interest (ROI). Collections of these quantitative image descriptors can be interrogated for statistical relationships with metrics or outcome measures relevant to a specific clinical use case and a cohort of patients.

This paradigm has been confirmed repeatedly in numerous studies across different imaging modalities, clinical application domains, and prediction tasks [3]. Despite its potential to revolutionize the development of personalized decision support models from standard-of-care imaging across clinical specialties, the translation of radiomics prediction models into clinical practice faces major challenges, many of which are linked to the quality and reporting of radiomics studies and affect the reproducibility of their findings [4].

Radiomic analysis is a complex multistep process [5] that entails the selection of clinical variables and imaging data, extraction of quantitative image descriptors, exploratory analysis of these descriptors, followed by the development and evaluation of predictive models to address a specific clinical question. This process relies on expertise from multiple disciplines, including image processing for feature extraction, machine learning for the training and evaluation of statistical models, medical physics for assessing the suitability of imaging protocols, and medical experience for interpreting the model and ensuring its clinical relevance. The heterogeneous backgrounds of radiomics researchers are reflected in the quality of the various steps performed and reported in radiomics studies [6, 7]. Consequently, standardization on different levels, including data collection, model evaluation, and reporting, has been identified as critical requirements for radiomics to mature as a discipline [8]. Indeed, progress toward community standards is underway and beginning to promote the homogenization of feature extraction approaches and algorithms [9, 10] as well as the reporting of radiomics studies in textual [8] and computable [11] form. “How-to” guides, such as those available in the literature [5, 12], are beginning to emerge that inform radiomics novices about common methodological caveats and propose practice-informed solution strategies.

While these developments increasingly improve the comparability of features and results across radiomics studies, many studies continue to lack methodological rigor or fail to demonstrate the potential clinical utility of their proposed prediction models. A recent review of radiomics studies in the field of neuro-oncology [13] indicates high adherence (> 80%) to the recommendations of the radiomics quality score [8] for the imaging protocol as well as for feature selection and validation but very mixed levels of adherence (approximately from 10 to 100%) for various aspects of model performance evaluation. The lowest adherence rates were observed for phantom and test-retest studies (from 0 to 2%), reporting of the potential clinical utility of the developed models (2%), and for providing high-level of evidence in the form of prospective studies (4%) or cost-effectiveness studies (0%).

Besides standardization of all aspects of radiomics analyses, translation of their results into clinical practice also requires adequate integration into the clinical workflow [5]. Physicians, the targeted end users of radiomics prediction models, often lack hands-on experience with this emerging technique and thus a realistic sense of its strengths and limitations [6]. However, physicians are best placed to formulate medically informed hypotheses that can link disease-related physiopathological events to radiomics feature categories. Despite their critical role as domain experts, physicians are rarely leading the development process of radiomics models, which might explain the lack of rigorous evaluation and demonstrated potential clinical utility in the majority of radiomics studies [13]. For radiomics to become the “bridge between medical imaging and personalized medicine” [8], we believe that physicians must be empowered to play a central role in the radiomics model development process.

Well-designed software and toolboxes that implement and enforce relevant standards have the potential to significantly lower the effort and technical expertise needed by application domain experts to perform complex analytical tasks [14]. We expect the availability of radiomics tools that provide a suitable level of abstraction and workflow integration to enable clinical domain experts to investigate their research questions using radiomics approaches. Furthermore, we hypothesise that interactive feature exploration and selection based on clinical knowledge and physiopathological hypotheses will lead to better interpretable and more generalizable models in the long term.

In this work, we propose a new vision of physician-centered radiomics research. We identify a minimum set of functional requirements for radiomics software to optimally support physician-centered radiomics research and comprehensively review existing radiomics tools and frameworks according to those criteria. Finally, we present our fully functional and openly accessible platform called *QuantImage v2*, which addresses the gaps identified among current radiomics tools and provides a prototype implementation of our physician-centered vision for radiomics research.

## Methods

**Fig. 1:**
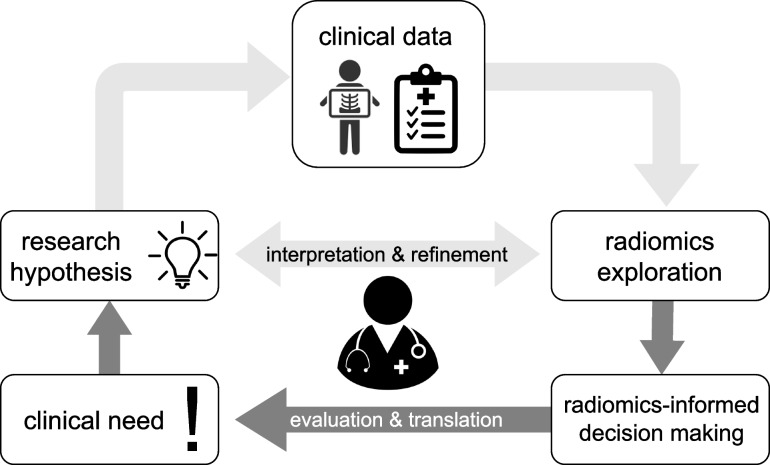
Physician-centered radiomics research. Physician-centered radiomics envisions medical doctors at the center of the radiomics research, development, and translation cycle

We believe that physicians play a critical role in the development of better interpretable and more generalizable radiomics prediction models and thus in the translation of radiomics research into clinical practice. Figure 1 illustrates this vision; as domain experts and medical practitioners, physicians are optimally positioned to develop and test radiomics research hypotheses aligned with real-world clinical needs. However, their participation in radiomics research is limited due to insufficient tooling support.

To achieve the vision of physician-centered radiomics research, software solutions must easily integrate into the hospital imaging data and information technology infrastructure. This implies that Digital Imaging and COmmunications in Medicine (DICOM) images with ROI annotations performed on clinical imaging software should be usable directly by the radiomics software and ideally be accessible from everywhere within the hospital network. We consider a web-based front end to be the most suitable entry point for such a system. Furthermore, we argue that the scope of radiomics tools must go beyond feature computation to optimally support radiomics research in a clinical setting; the software should guide the user through the radiomics analysis workflow and enable cohort management, interactive feature exploration, and selection, as well as training and validation of prediction models, all without the need for programming knowledge. To support users without prior experience in radiomics research, additional information should be provided, and the individual processing steps be facilitated through visualization, contextual help, automation, and meaningful defaults wherever possible.

Along with the increasing interest in radiomics research, multiple tools have been developed that address different aspects of the radiomics workflow. Previous works reviewed existing radiomics software with a focus on their feature extraction capability and assessed the comparability of features computed by different radiomics platforms [15–17]. To identify best practices and reveal the shortcomings of existing software solutions to optimally support physician-driven radiomics research in a clinical environment, we reviewed the functionality of existing “free-to-access” radiomics tools along five dimensions: (1) ease of use; (2) ability to handle different imaging formats; (3) support for population studies; (4) ability to extract, manage, and explore radiomics feature sets; and (5) ability to manage, train, and test prediction models.

Based on this review, we designed and implemented *QuantImage v2* [18], the successor of QuantImage [19], to embody the vision of physician-centered radiomics research.

## Results

### Existing tools for radiomics research

We identified 26 noncommercial radiomics tools, of which four were not publicly accessible at the time of writing [20–24]. Table 1 reports the 22 freely accessible (OpenSource, FreeWare) tools, and *QuantImage v2*, along the following dimensions:

- *Type*: Based on the skill level required for usage and accessibility, we distinguish between (a) programming libraries (*library*), (b) command-line tools (*cmd-exec*) that do not require specific programming skills, and (c) application frameworks that provide a graphical user interface (GUI), either as locally installed stand-alone applications (*GUI*), plug-ins into application frameworks (*GUI plug-in*), or web-based applications (*GUI web*) which can be accessed in a device-independent manner.
- *Imaging*: We distinguish tools that can work directly with DICOM images and annotation data from those that require prior conversion. Several DICOM-enabled tools provide functionality similar to a clinical picture archiving and communication system (PACS), which allows for image and annotation management. Also, some tools integrate support for image *segmentation*.
- *Cohort*: The ability to manage feature and outcome variables of multiple patients is an essential prerequisite for cohort-based visualizations, statistical analysis, and thus prediction modelling. Few tools provide such functionality, either by consuming files (*file*) or as part of a web interface (*web based*).
- *Radiomics features*: All considered tools provide functionality for feature extraction, some conforming to the recommendations of the imaging biomarker standardization initiative (IBSI) [9]. We distinguish tools without feature visualization capability from those that can visualize spatial feature maps of a single region of interest (*feature map*) and those that can visualize the values of collections of features for a given patient cohort (*cohort*). Feature selection before the model building is an important step to avoid overfitting; however, cohort-level feature visualization and selection are reserved for tools that support cohort management.
- *Prediction modelling*: Similarly, training and evaluation of prediction models via statistical and machine learning require the respective tools to support the notion of a patient cohort and derived collection of features. Model management refers to functionality for remembering and comparing prediction models, including their performance and provenance information.

**Table 1:** Three major groups of radiomics tools were identified: (top to bottom) programming libraries and command-line executables (typically) limited to a narrow set of functionalities focused on radiomics feature extraction; toolkits bundling multiple functionalities for radiomics workflows; advanced applications providing additional functionality for radiomics analyses

Name	Type	Imaging		Cohort	Radiomics features			Prediction modeling		
		DICOM	Segmentation	Management	Extraction	Visualization	Selection	Training	Evaluation	Management
**PyRadiomics**	Library	--			X^(c)^					
**moddicom**	Library	X			X					
**RADIOMICS**	Llibrary	X			X					
**PORTS**	Library	--			X					
**ROdiomiX**	cmd-exec	X			X^(d)^					
**SERA**	Library/cmd-exec	--			X^(c)^					
**QIFE**	Library/cmd-exec	X			X^(c)^					
**MIRP**	Library	X			X^(c)^					
**RaCaT**	cmd-exec	X			X					
**Precision-medicine-toolbox**	Library	X		txt/xlsx file	PyRadiomics	Cohort		X	X	
**LIFEx**	GUI^(a)^	X^(b)^	X		X^(c)^	Feature map				
**IBEX**	GUI	X	X		X	--				
**CERR**	GUI (plugin)	X	X		X^(c)^	Feature map				
**MRP**	GUI (plugin)	X	X		X	--				
**MITK Phenotyping**	GUI (plugin)	X	X		X^(c)^	--				
**SlicerRadiomics**	GUI (plugin)	X	X		PyRadiomics	--				
**CGITA**	GUI (plugin)	X^(b)^	X		X	--				
**QuantImage (v1)**	GUI (web)	X^(b)^	--		X	--				
**ePAD**	GUI (web)	X^(b)^	X		QIFE	Feature map				
**MaZda/b11**	GUI	--	X	txt file	X	Feature map	Automated	X	--	--
**CaPTk**	GUI	X^(b)^	X	txt file	X^(c)^	Feature map	--	X	X	Folder based
**AutoRadiomics**	GUI (web)	X	X	txt file	X^(c)^	Feature map	Automated	X	X	X
**QuantImage v2**	GUI (web)	X^(b)^	--	Web based	X^(c)^	Cohort	Interactive	X	X	X

Cross (X) or dashes (--) indicate the presence or absence, respectively, of a specific characteristic; features not typically applicable to a specific tool category are left empty. (a) Free-ware instead of open-source, (b) PACS-like functionality, (c) Participation in the IBSI benchmark [9], (d) Self-reported adherence to IBSI recommendations. *IBSI*, Image Biomarker Standardisation Initiative, *PACS*, Picture Archiving and Communication System

Based on their type and functionalities, we identified three major groups with the following characteristics:

- Programming libraries and command-line executables (PyRadiomics [25], moddicom [26], RADIOMICS [27], PORTS [28], ROdiomiX [29], SERA [30, 31], QIFE [32], MIRP [33, 34], RaCaT [35], precision-medicine-toolbox [36, 37]) that typically focus on a narrow set of functionalities, usually feature extraction, and require programming skills for their usage or inclusion of the generated data in the radiomics analysis workflow
- Toolkits that bundle multiple basic functionalities needed specifically for radiomics research (LIFEx [38], IBEX [39, 40], CERR [41, 42], MRP [43], MITK Phenotyping [44], SlicerRadiomics [45], CGITA [46], QuantImage [19], ePAD [47]). These are often integrated into popular medical imaging applications (*e.g.*, 3D Slicer [48], MITK [49]) or general-purpose data analysis environments (MATLAB [50]). They perform feature extraction and export through a GUI and may provide image viewing and segmentation functionality. However, they rarely support the exploration of radiomics features across a patient cohort or the development/evaluation of prediction models.
- Radiomics applications (MAZDA [51, 52], CaPTk [53, 54], AutoRadiomics [55, 56]) that provide the same features of (b) as well as some modelling capability in a dedicated stand-alone or web-based application

Most existing radiomics tools do not provide the level of abstraction needed to make radiomics research easily accessible for experts without specialist knowledge in data mining and modelling. While most tools included in our review (see Table 1) allow feature extraction from a single image or a collection of images, subsequent building, and evaluation of radiomics models typically requires another library or tool for model building to be used, thus disrupting the analysis workflow.

Separate libraries and software solutions exist for exploring and modelling with high-dimensional data; however, only few software solutions support the entire radiomics research workflow by integrating functionalities for both radiomics feature extraction and population modelling. Only a small subset of tools supports the management of patient cohorts or associated outcome variables. MAZDA [51, 52], CaPTk [53, 54], AutoRadiomics [55, 56], and to a lesser extent the precision-medicine-toolbox [36] provide functionality for associating outcome values to radiomics features derived from multiple images or ROIs, thus enabling the development of prediction models. ePAD [36] relies on the Quantitative Image Feature Engine, QIFE [36], and the Quantitative Feature Explore, QFExplore, Plugin Suite [57] for feature extraction and exploration. However, these tools lack advanced visualization and model management capabilities that would facilitate iterative model development and hypothesis testing. Integrating data and model management facilities with other radiomics and modelling specific functionalities are essential for cohort-based radiomics analysis and thus a key requirement for a physician-centered radiomics software system. The AutoRadiomics framework [55] provides the most complete feature set according to the dimensions defined in Table 1. However, while its user interface is browser based, input and output management via file paths requires local installation and would prevent its shared use and data management by multiple collaborators.

### QuantImage v2: a tool for physician-centered radiomics research

QuantImage v2 (QI2) was designed and implemented to address these shortcomings. Rather than developing individual components from scratch, QI2 combines well-established existing tools and open-source libraries to realize and concretely demonstrate the potential of a one-stop tool for physician-driven radiomics research.

**Fig. 2:**
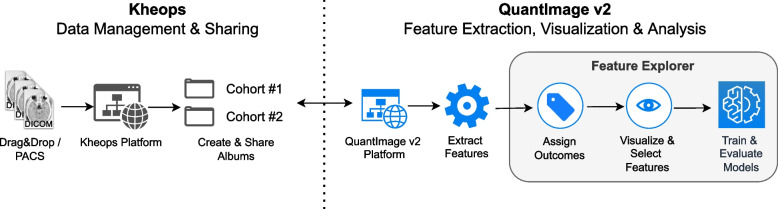
Functional and technical components of the proposed physician-centered radiomics research platform. The QuantImage v2 (QI2) platform relies on Kheops for image management and visualization. QI2 provides web-based graphical user interfaces for feature extraction, feature exploration, visualization, and radiomics model development and validation

QI2 provides web-based access to cohort management, feature extraction, and visualization and supports the development and evaluation of machine learning models against patient-specific clinical or outcome data. For the management of DICOM collections, QI2 relies on the Kheops platform [58], a PACS-like system with a web-based portal for upload, download, organization, sharing, and visualization of DICOM images and segmentations (radiotherapy structure set, RT-STRUCT; DICOM segmentation object, DICOM-SEG). Kheops can receive images directly from the clinical PACS or via an intermediate DICOM-web compatible anonymization service such as Karnak [59]. Figure 2 summarizes the functional components of Kheops and QI2; arrows indicate the typical sequence of user interactions, starting from the curation of an image collection in Kheops, to feature extraction, the input of clinical or outcome data, visual feature exploration, and training and evaluation of machine learning models.

### QI2 functionalities

**Fig. 3:**
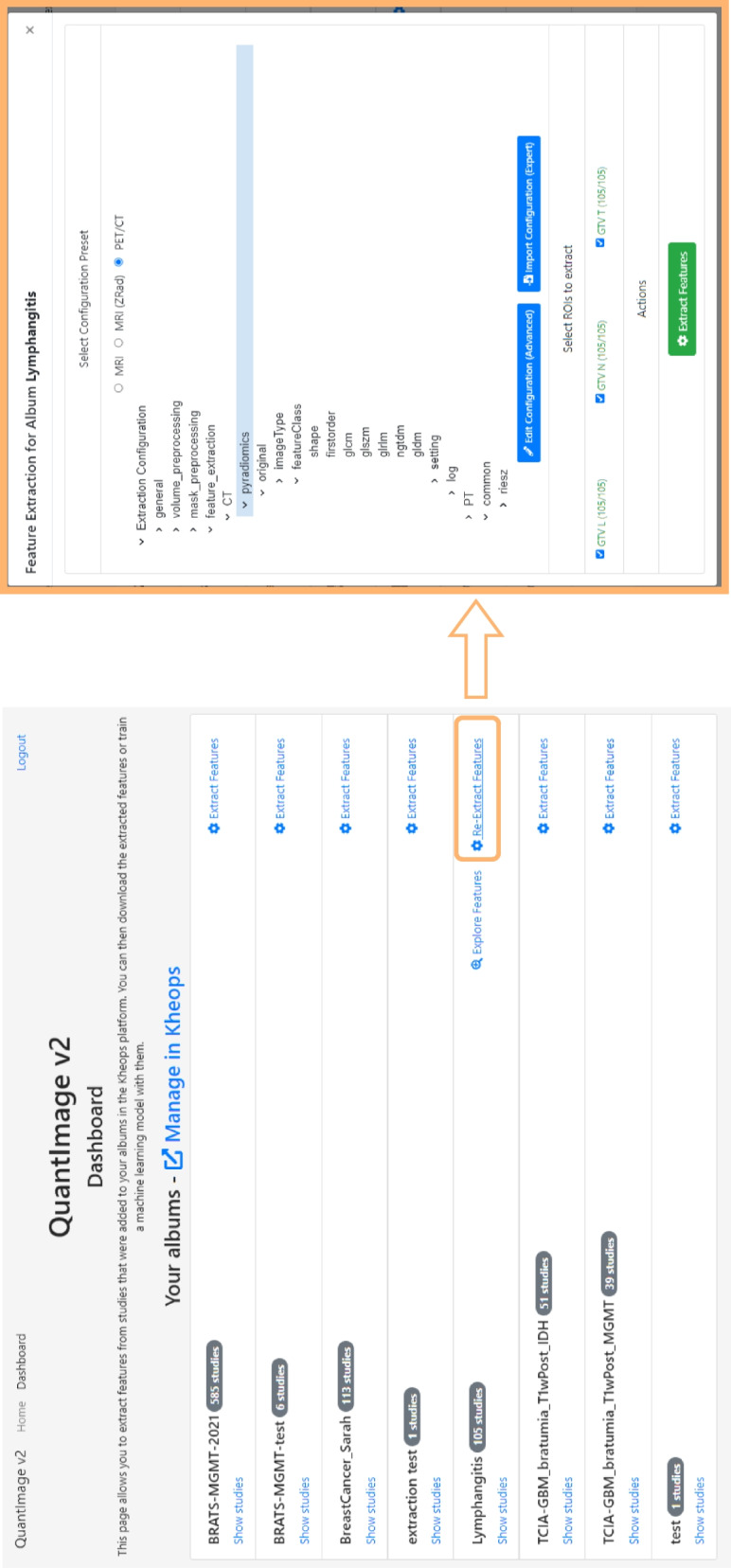
QuantImage v2 (QI2) dashboard and feature extraction dialogue. The QI2 *dashboard* (left) provides an overview of the user’s Kheops image collections and gives access to the *Feature Extraction* and the *Feature Explorer* (see Fig. 4) interfaces. The *Feature Extraction* interface (right) permits selecting the ROIs and choosing from default extraction configurations for PET/CT and MRI image collections. Advanced users can control all aspects of the feature extraction process by editing the detailed configuration parameters exposed by the interface. *PET/CT*, Positron emission tomography/computed tomography; *MRI*, Magnetic resonance imaging; *ROI*, Region of interest

Once logged in, the user is presented with all accessible Kheops image collections, *i.e.*, patient cohorts. A dedicated *Feature Extractor* screen allows configuring the main aspects of feature extraction (Fig. 3). First, the user can choose among several presets for feature extraction, including the feature types to be extracted, as well as the libraries (*e.g.*, PyRadiomics [25] or Riesz [60]), and image pre-processing settings (*e.g.*, resampling, standardization for magnetic resonance images) to be utilized. Second, all ROIs are gathered from the segmentations of the imaging dataset so that the user can select the ROIs from which features are to be extracted. An indicator informs the user about the progress of feature extraction, and all computed features are stored in a database for future reuse.

**Fig. 4:**
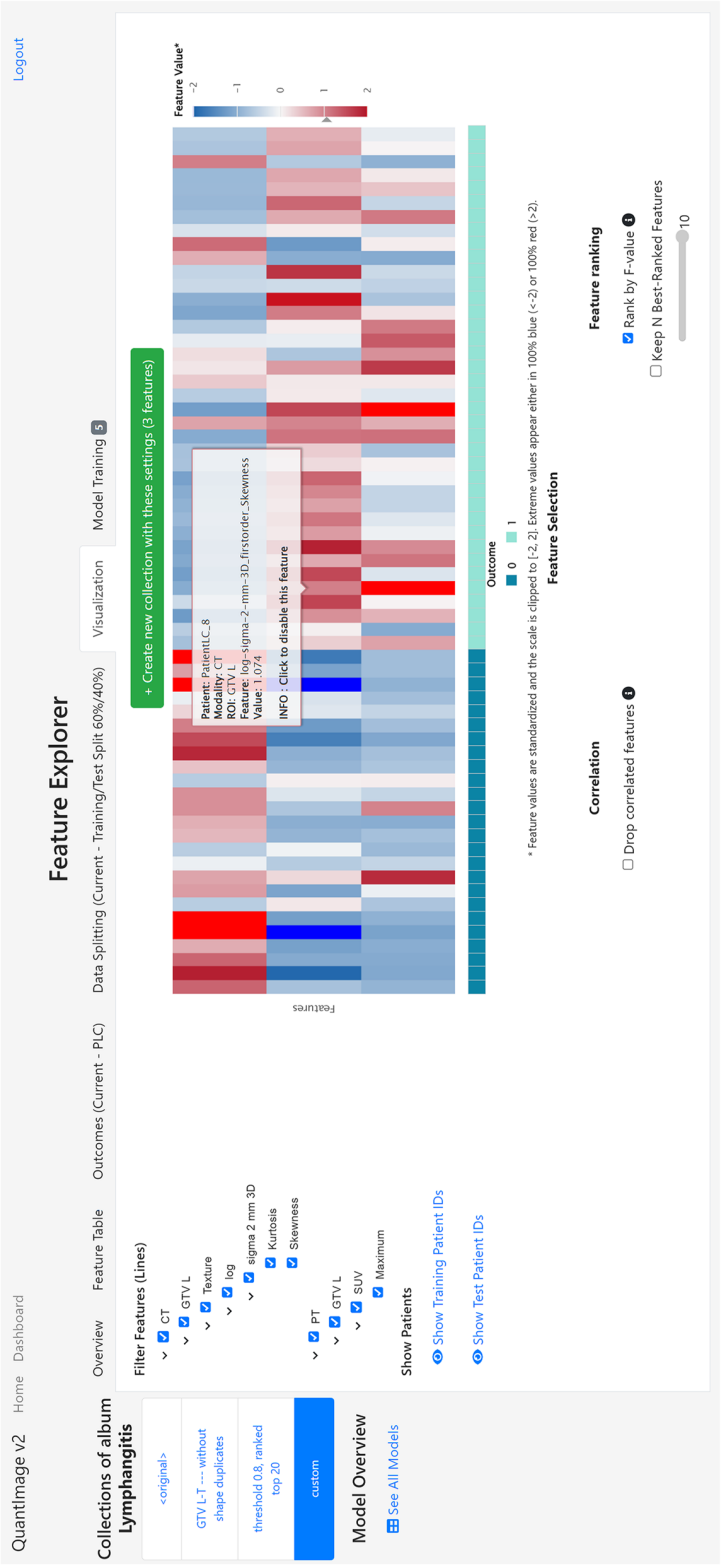
QuantImage v2 (QI2) feature explorer and visualization. The *Visualization* tab of the QI2 *Feature Explorer* provides feature selection and visualization functionalities. A tree-like filter mechanism allows selecting specific features based on the imaging modality (*e.g.*, PET, CT) and ROI (here: “GTV L”) from which they were derived and grouped by feature class (*e.g.*, “texture”). A heatmap visualizes the values of the selected features for all chosen patients ordered by the outcome measure. Automated feature selection and ranking options can be applied to refine this view. The three selected features displayed here (CT kurtosis, CT skewness derived from Laplacian-of-Gaussian filtered images, and PET SUVmax) are highly predictive of the presence of pulmonary lymphangitic carcinomatosis (average area under the curve of 0.94 and 0.88 from cross-validation and on test set, respectively). *PET*, Positron emission tomography; *CT*, Computed tomography; *ROI*, Region of interest; *SUV*, Standardized uptake value

**Fig. 5:**
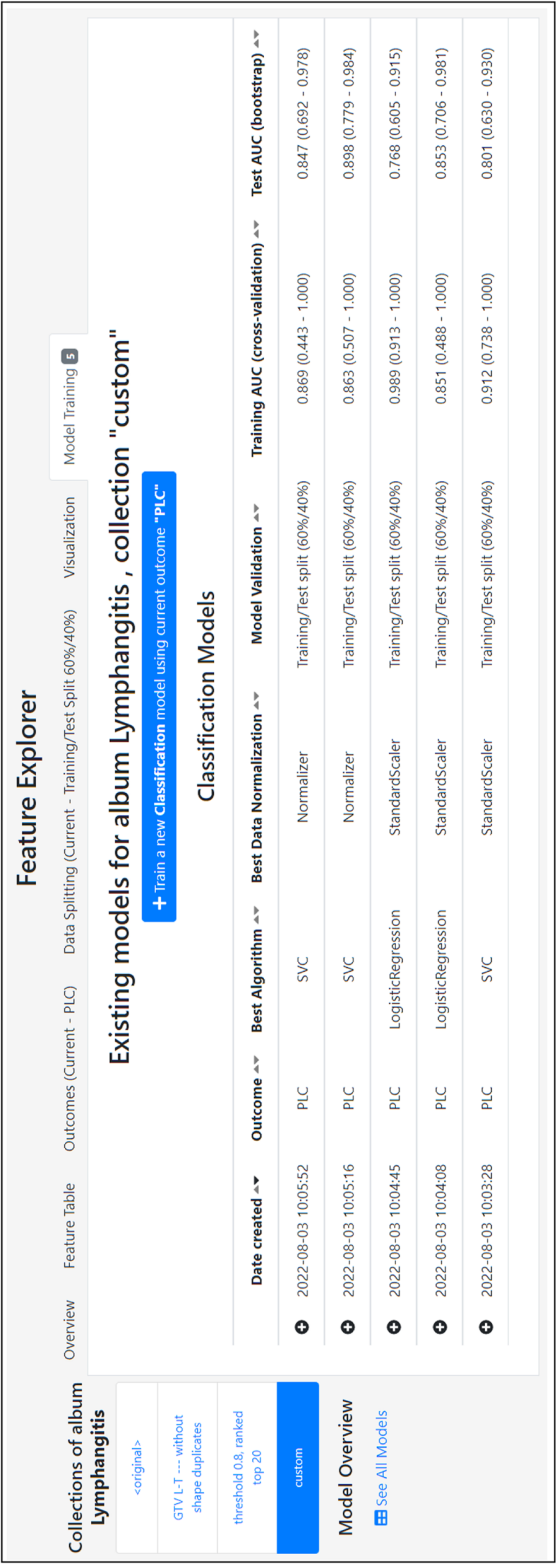
QuantImage v2 (QI2) feature explorer and model training. The Model Training tab allows triggering machine learning model building and validation. It also provides a summary table listing all existing models trained against a specific feature collection, along with their characteristics

The user can explore the extracted features and interact with them in several ways using the *Feature Explorer*. This interface provides the following: (a) an overview of the types of features that have been extracted, *e.g.*, modalities, ROIs, number of patients, and number of features, (b) a tabular view allowing inspection of all extracted feature values, (c) upload of patient-specific outcomes, and (d) a data splitting functionality to choose between visualizing either the entire data or leaving out a test set for further validation of trained models. The iterative feature selection process is realized via (e) a visualization interface in which all or a subset of features can be explored via heatmaps (Fig. 4), and (f) a model training interface (Fig. 5), in which machine learning (ML) models can be trained and evaluated using established ML libraries (*e.g.*, *scikit-learn* [61], *scikit-survival* [62]).

### QI2 support for radiomics studies workflow

In addition to performing end-to-end radiomics analyses over clinical data, QI2 provides functionalities to support iterative radiomics workflows. Frequently, a user may wish to test several hypotheses using various subsets of features, *e.g.*, comparing radiomics models based on different imaging modalities or feature groups. To achieve this, QI2 allows for the creation and management of *feature collections*, *i.e.*, custom sets of features extracted from specific imaging modalities and/or ROIs. Each collection can be used as the basis for more refined visualization and predictive model training using only the subset of features contained within that collection. Advanced visualization functionalities and hierarchical filtering facilitate the selection of salient feature groups into these custom feature collections and to identify outlier patients. Furthermore, QI2 provides automated feature selection strategies that allow suppressing strongly correlated features and selecting the top *N* univariately most predictive features based on the analysis of variance, ANOVA, and F-statistic, where outcome subgroups are considered (Fig. 4).

QI2 supports the training, evaluation, and management of different types of machine learning models for classification and survival analysis on these feature collections. For a chosen outcome variable, feature collection, and train/test split, QI2 identifies the optimal prediction model by performing a “grid search” (5-fold cross-validation on the training set) over many candidate models (features standardization approaches, ML algorithms, parameter sets). The best performing model is evaluated on the test set via bootstrapping, and its characteristics and performance are reported in an overview table that summarizes the outcomes of all trained models, as illustrated in Fig. 5. Additional details, such as the ML algorithm, selected modalities, and features, or further performance metrics, can be accessed by clicking on one of the model entries.

### A use-case: radiomics diagnostic model of pulmonary lymphangitic carcinomatosis (PLC)

We illustrate the use of QI2 for developing a diagnostic model of PLC, a condition linked to very poor prognosis in the context of non-small cell lung cancer (NSCLC) and defined by the invasion of the lymphatic system by cancerous cells. Despite being associated with subtle increased peritumoral uptake in ^18^F-FDG PET as well as peribronchovascular thickening in CT images, diagnosing PLC on high-resolution CT remains a very difficult task for human readers, especially in subtle cases [63]. The current reference standard for final diagnosis is the histopathological analysis of the resected lung. Hence, less invasive methods that can provide more information on PLC, before the surgical intervention, would greatly help clinicians identify the best treatment.

A collection of 105 cases, among which 64 (61%) patients were diagnosed with PLC, was curated at the Lausanne University Hospital (CHUV) as approved by the local ethics committee (CER-VD 2018-01513). An ^18^F-fluorodeoxyglucose (FDG) positron emission tomography (PET)/CT scan was performed on all patients as part of the initial staging of NSCLC, and an expert radiologist contoured two types of ROIs: the tumoral (ROI-T) and the peritumoral, aimed at identifying the potential lymphangitis (ROI-L). The ROI-T was defined as the tumor itself. The ROI-L was a spheroidal volume of approximately 3 cm^3^ placed in the vicinity of the tumor where an abnormal FDG uptake was observed.

Using QI2, radiomics features were extracted from both ROI-L and ROI-T regions. QI2’s *Feature Explorer* was used to identify the features being most predictive of PLC. An initial model based on all 800 radiomics features from ROI-L and ROI-T led to an average cross-validated area under the curve AUC of 0.89 (average AUC from bootstrapping on test set: 0.77) across five different dataset splits (60% cross-validation, 40% test). QI2’s automatic feature selection tools helped identify a more predictive subset of features by removing correlated and selecting the 20 individually most predictive features. The resulting model yielded an average cross-validated AUC of 0.94 (average AUC from bootstrapping on test set: 0.88). Models using a manually selected subset of ROI-L features (maximum standardized uptake value, SUV_max_, and two Laplacian of Gaussian (LoG)-based CT texture features, Fig. 4) that had previously been found to be predictive of PLC [64] yielded an average cross-validated AUC of 0.90 (average AUC from bootstrapping on test set: 0.83) (Fig. 5), thus confirming the importance of this imaging signature.

## Discussion

While many studies demonstrated the potential of radiomics for personalized oncology, every combination of the trio (i) disease, (ii) imaging modality, and (iii) clinical endpoint requires a full investigation of its own. Despite the availability of many radiomics tools, no existing platform supports the entire radiomics model-development cycle in a way that integrates well into the clinical data workflow and is easily accessible to clinical domain experts.

We identified essential and desirable characteristics of such a platform and propose QuantImage v2 (QI2) as a prototype implementation. We believe that physician-centered radiomics research with QI2 will improve the quality of radiomics studies by addressing several criteria of the radiomics quality score (RQS) [8]. RQS evaluates a radiomics study along multiple dimensions (RQS criteria) by assigning points for a given criterion; a higher total RQS corresponds to better quality radiomics study. In the case of QI2, feature reduction techniques (RQS criterion 5, +3 points) are readily available, and discrimination statistics (RQS criterion 9, +2 points) such as AUC are computed using resampling methods (cross-validation, bootstrapping). Basic validation on an unseen subset of the data collection (RQS criterion 12, at least +2 points) is performed automatically. Furthermore, the paradigm of physician-centered radiomics research intrinsically features a strong focus on the potential clinical utility (RQS criterion 14, +1 point) of developed radiomics models and on explaining radiomics signatures in terms of the underlying biology and physiology (RQS criterion 7, +1 point). QI2 and similar tools can make the radiomics processing workflow more reproducible, thus providing unique opportunities for facilitating larger scale model validation on external datasets (RQS criterion 12) and Open Science (RQS criterion 16).

While QI2 features what we consider the essential functionalities for supporting a clinician-centered radiomics workflow, various limitations remain: QI2 relies on existing contours, it does not currently support radiomics modelling involving multiple imaging timepoints (so-called delta-radiomics), nor allow for the extraction of features via deep-learning (so-called deep features) or handle other “omics” as features data. QI2 uses carefully chosen defaults to yield robust results for a variety of radiomics tasks, but its current implementation does not provide fine-grained control of model training and evaluation settings. However, advanced users can download the extracted features for integration in their custom machine-learning pipelines. Finally, due to the quick turnaround between feature selection and model training and evaluation, users of QI2 may be tempted to select an “optimal” feature set based on the test performance of multiple successively refined models. QI2 leaves the responsibility of recognizing and mitigating the resulting risk of overfitting with the user.

To further evaluate our implementation and its underlying design choices for physician-driven radiomics research and education, a user study is being performed in collaboration with the Department of Nuclear Medicine Department of Nuclear Medicine and Molecular Imaging at the Lausanne University Hospital, Switzerland. Further information about QI2 is available at https://medgift.github.io/quantimage-v2-info/, including access to a virtual machine image for local (*e.g.*, sensitive data) deployment and testing, as well as to the QI2 source code. A deployed instance providing access to the training data of the 2020 HECKTOR challenge [65] can be accessed at https://quantimage2.ehealth.hevs.ch/.

## Data Availability

A publicly deployed instance of QI2 with access to the training data of the 2020 HECKTOR challenge [65] can be accessed at https://quantimage2.ehealth.hevs.ch/; a virtual machine image for local (*e.g.*, sensitive data) deployment and testing, as well as the QI2 source code, are available at the QI2 website https://medgift.github.io/quantimage-v2-info/. The dataset used for illustrating the functionality of QI2 in this manuscript cannot be made publicly available due to legal constraints.

## Abbreviations

AUC: Area under the curve
CT: Computed tomography
DICOM: Digital Imaging and COmmunications in Medicine
FDG: Fluorodeoxyglucose
GUI: Graphical user interface
IBSI: Image Biomarker Standardisation Initiative
ML: Machine learning
NSCLC: Non-small cell lung cancer
PACS: Picture archiving and communication system
PET: Positron emission tomography
PLC: Pulmonary lymphangitic carcinomatosis
QI2: QuantImage v2
ROI: Region of interest
ROI-L: Peritumoral ROI with potential lymphangitis
ROI-T: Tumoral ROI
RQS: Radiomics quality score
